# Health Inequalities in the Time of COVID-19: The Globally Reinforcing Need to Strengthen Health Inequalities Research Capacities

**DOI:** 10.1177/0020731421993939

**Published:** 2021-03-08

**Authors:** Lucinda Cash-Gibson, Juan M. Pericàs, Eliana Martinez-Herrera, Joan Benach

**Affiliations:** 1 Research Group on Health Inequalities, Environment and Employment Conditions, 16770 Pompeu Fabra University, Barcelona, Spain; 2 Pompeu Fabra University Public Policy Center, Johns Hopkins University, Barcelona, Spain; 3 16493 Hospital Clínic de Barcelona, Barcelona, Spain; 4 Institute for Research, 16810 Vall d’Hebron Hospital Universitari, Barcelona, Spain; 5 Research Group of Epidemiology, National School of Public Health “Héctor Abad Gómez”, 27983 University of Antioquia, Medellín, Colombia; 6 Transdisciplinary Research Group on Socioecological Transitions (GinTrans2), 16722 Universidad Autónoma de Madrid, Madrid, Spain

**Keywords:** COVID-19, health inequalities, research capacity strengthening, health policy

## Abstract

The full impact of coronavirus disease 2019 (COVID-19) is yet to be well established; however, as the pandemic spreads, and early results emerge, unmet needs are being revealed, and pressing questions are being asked about who is most affected, how, where, and in what ways government responses might be exacerbating inequalities. A number of scholars have called for more in-depth critical research on COVID-19 and health inequalities to produce a strong empirical evidence based on these issues. There are also justifiable concerns about the scarcity of health-equity actions oriented analyses of the situation and calls for more empirical evidence on COVID-19 and health inequalities. A preliminary condition to establish this type of information is strong capacity to conduct health inequalities research. Worldwide, however, this type of capacity is limited, which, alongside other challenges, will likely hinder capacities of many countries to develop comprehensive equity-oriented COVID-19 analyses, and adequate responses to present and future crises. The current pandemic reinforces the pending need to invest in and strengthen these research capacities. These capacities must be supported by widespread recognition and concern, cognitive social capital, and greater commitment to coordinated, transparent action, and responsibility. Otherwise, we will remain inadequately prepared to respond and meet our society’s unmet needs.

While the full health, social, and economic impacts of coronavirus disease 2019 (COVID-19) are yet to be established, firstly, evidence from past pandemics, nature diseases, and crises show that the most “socially vulnerable” (ie, those with the lowest social positions and power due to structural factors and systematic, unfair, and avoidable social hierarchies^1^) are often disproportionately exposed, prior, during, and after such devastating events.^2–9^ Also, early results from a number of countries and cities have identified that social inequalities exist in the disease incidence and mortality from COVID-19.^2,3,10–18^ As the pandemic spreads, pressing new questions arise that need to be understood, such as why do certain social groups appear to be more affected than others? What are the causes of these systematic differences? What is the effect and impact of current policy responses on preexisting health inequalities between and within populations in different global contexts? What will appropriate, equitable responses look like?

## Comprehensive Health-Equity Oriented Analyses: What? Where? How? Why?

Globally, there is a paucity of health-equity oriented analyses and policy reactions to the pandemic, which limits the capacity to determine how, and in which ways, government responses are impacting, and potentially exacerbating existing health inequalities. A number of scholars have called for more in-depth critical research on COVID-19 and health inequalities, to produce strong empirical evidence based on these issues.^3,8,11^ To build a comprehensive and integrated picture of the causes, distribution, and impact of the pandemic on society, requires having a strong capacity to monitor, analyze, and conduct equity-oriented COVID-19 research, which includes transparent collection of available and reliable health data, disaggregated by social groups and geographical territories, and on environmental, social, economic, political, and labor determinants.^2,8,19^ Another imperative is a critical mass of motivated professionals, trained in multiple disciplines, to recognize and conceptualize the health inequality problems, test and generate theories, map data sources, and capable of analyzing, interpreting, and reporting the results^19^; all of which should be supported by adequate stewardship, leadership, and resource provision.^19–22^ Within the city of Barcelona, for example, several institutions have been able to rapidly identify that social inequalities exist in the disease incidence and mortality from COVID-19 at the neighborhood level.^10,12,13^ This particularly strong capacity to generate evidence on health inequalities at the city level is partly due to a long tradition of social recognition and concern for public health and social injustice, and solid preexisting multidisciplinary research capacity at the individual, research infrastructure, and institutional level.^23^ Also, continuous access to available, reliable, and transparent data collection and reporting, and the more recent development of an observatory for public health and inequalities, and political prioritization to reduce health inequalities,^24^ amongst other things.^23^

Worldwide, however, many countries still do not have a strong capacity to conduct scientific evidence on health inequalities, to effectively inform efforts aiming to address their population's health and well-being needs.^19,23,25,26^ For example, a number of countries, particularly low- and middle-income ones, still face huge challenges in terms of health and geographical information and surveillance resources, regarding their completeness and quality, as well as in the availability of reliable, disaggregated, and integrated health and socio-demographic data, to support the measuring and monitoring of health inequalities,^19^ as recently illustrated by the cases of Ecuador^26,27^ and Mozambique.^28^ Although census data collected in high-income countries, such as in the United States, are not necessarily free from problems.^29^ Collectively, these challenges will hinder many countries’ capacities to comprehensively prevent, analyze, and manage current and future problems, such as racial inequalities, particularly during the pandemic, as recently highlighted in the case of Brazil,^26^ as well to design and implement effective and equitable responses to address them, and to monitor and evaluate their impact on the (entire) population. This is on top of the fact that many countries, particularly lower-income ones, face resource shortages which will also inhibit effective COVID-19 preparedness.^30,31^

## Research Capacity Needs and Challenges

The current pandemic reinforces the unmet need to invest in and strengthen health inequalities research capacity in many local and global settings. We agree with the increasing number of calls to gather much-needed disaggregated data by socio-demographic and socio-economic characteristics of people infected and affected by COVID-19.^2,32–34^ Yet, at the same time, to fully understand the causes of the systematic social differences in COVID-19-related infection and mortality (eg, by gender, race/ethnicity, socio-economic status, migrant status, social class, etc), will require a balanced gathering of data on biomedical features, individual choices, and behaviors,^2^ and information on social determinants of health, including unjust living situations, work conditions, educational provisions, power relations, and social mechanisms (eg, exploitation, discrimination, racism, and sexism) that shape material circumstances, behaviors, and biology. As well as a better-contextualized understanding of the process of disease embodiment, that is the complex biological effects of the bio-psycho-social–environmental interplay to produce health inequalities,^35^ and the cumulative impact of socio-economic adversity, political marginalization, in different settings.^2,19,36^ Furthermore, conducting health inequalities research capacity assessments at the local/national level could help identify pending challenges, barriers, and information gaps, and assist to guide future strategies that aim to address their specific capacities.^19,37^

We summarize some of the potential challenges that will likely need to be addressed in different contexts, in order to strengthen their capacity to generate health equity-oriented COVID-19 research, which can be used to inform appropriate and equitable responses^23^ (see Table 1).

**Table 1.** Potential Health Inequalities Research Capacity Challenges to Consider in Different Contexts.

Level	Potential health inequality research capacity challenges to address
Global	Limited stewardship, governance, and lack of human and financial and technical resources^19,21^; limited demand for, and prioritization of, locally relevant health equity-oriented COVID-19 research^19,21^; conflicting socio-political value judgments, ideology, and interests around health inequalities, and fair and inequitable responses between countries and institutions.^19,23,38,39^
National, regional, or city	Limited stewardship, governance, and limited provision of health inequalities related human and financial and technical resources^19,21,37^; limited tradition of public health and health inequalities research^37^; conflicting socio-political value judgments, ideology and interests around health inequalities, and fair and inequitable responses between institutions and stakeholders^19,37^; limited demand for, and prioritization of locally relevant health equity-oriented COVID-19 research^19,21,28^; limited demand for available, transparent and reliable, disaggregated and integrated health and socio-demographic data^12,13,37^; limited academic freedom or creative autonomy to reflect, propose and pursue critical research on global-societal-health issues, such as health inequalities, particularly during pandemics.^19^
Institutional	Limited stewardship, limited provision of human, financial and technical resources, facilities, and infrastructure^19,21,40^; conflicting social-political value judgments, ideology and interests^19,38,39^; limited demand for, and prioritization of, locally relevant health equity-oriented COVID-19 research^19,21^; limited academic freedom or creative autonomy to reflect, propose, and pursue critical research on global-societal-health issues such as health inequalities, particularly during pandemics.^19^
Research infrastructure: Information systems	Limited stewardship, governance, and limited provision of human, financial, and information resources^19,21,40^; limitations in the completeness and quality of geographical information and surveillance resources, with a health equity lens^27,28,37^; limitations in the available, reliable, disaggregated and integrated health and socio-demographic data, to support the measuring and monitoring of health inequalities.^2,19,27,28,32,37,40^
Research infrastructure: Human resources	Limited access to available training in integrating transdisciplinary perspectives to be able to understand, analyze, and monitor health inequalities^19,21,37,40^; “brain-drain”^19,21^; lack of a local critical mass of transdisciplinary professionals, trained to understand, analyze, monitor, and evaluate health inequalities, and other complex global-societal issues such as pandemics^19,21,37^; conflicting socio-political value judgments, ideology, and interests among research groups and individual researchers.^19,23,37–39^
Research networks	Limited pooling and mobilizing of (local and international) resources and cognitive social capital to co-develop effective solutions to address health inequalities during complex global–societal times^19,21,23,37^; conflicting socio-political value judgments, ideology and interests among research groups and individual researchers.^19,23,37–39^
Research output	Limited volume of health equity-oriented COVID-19 analyses, and transparent reporting of locally relevant findings, published in peer-review academic international journals.^12,13,19,21,25,37^
Research usage	Challenges in communicating and disseminating these research findings to different audiences in an accurate, appropriate, and timely manner.^5,31^

## The Ways Ahead

Even in the absence of a comprehensive diagnosis, denial, ignorance, and inaction are not options.^7^ Underlying the decision and actions taken, or neglected, to address socially relevant issues, such as social inequalities in health, are divergent (political, social, and personal) values, ideological views, and tensions over what should be done, what is fair, and what is possible, as well as the type of research that should be funded and generated.^7,19,23,29,38,39^ These diverse perspectives become even more visible during times of crises and pandemics. Subsequently, the socio-political context in which public health and health inequalities research, and political priorities are planned and implemented, also need to be evaluated to better understand the causal forces and dynamics operating at different (macro-to-micro) levels, which influence and determine the capacity to act in an equitable manner.^19,23,37^

The increase in social inequalities in health, infectious disease outbreaks, and ecological changes over the past few decades are arguably symptoms of systemic dysfunctions, and incompatibilities with our decisions and choices in pursuit of progress and prosperity,^8,42^ which are adversely shaping and influencing our opportunities to lead healthy lives in the process.^7^ Going forward, widespread recognition, sensitivity, and concern for these problems need to be instilled, at the political, social, institutional, and individual level.^23,43,44^ Also, strong cognitive social capital (e.g., social norms related to solidarity, trust, and social participation^45–49^) must be fostered to co-produce innovative, effective, and equitable solutions for health to our increasingly globalized problems; both of which need to be supported by greater responsibility, cooperation, and commitment to coordinated, transparent action.^23^ Otherwise, we will continue to remain inadequately prepared to respond, manage, and address our society’s unmet needs.
